# Health Status and Use of Healthcare Services of Undocumented Migrant Sex Workers in Catalonia: A Descriptive Study Using Administrative Registries

**DOI:** 10.3390/ijerph22111696

**Published:** 2025-11-10

**Authors:** Albert Dalmau-Bueno, Sergi Albert-Ballestar, Rosa Mansilla, Anna García-Altés

**Affiliations:** 1 Catalan Agency for Health Quality and Assessment of Catalonia (AQuAS), 08009 Barcelona, Spain; alb.dalmau@gmail.com (A.D.-B.); albertsergi@gmail.com (S.A.-B.); 2 CIBER de Epidemiología y Salud Pública (CIBERESP), 28029 Madrid, Spain; 3 Programa de Prevenció, Control i Atenció al VIH, les ITS i les Hepatitis Víriques (PCAVIHV), Agència de Salut Pública de Catalunya, Departament de Salut, Generalitat de Catalunya, 08028 Barcelona, Spain; rosa.mansilla@gencat.cat; 4 Institut d’Investigació Biomèdica (IIB Sant Pau), 08041 Barcelona, Spain

**Keywords:** undocumented migrant sex workers, healthcare use, STDs, administrative registries, real-world evidence

## Abstract

The objective of the study is to describe the health status and use of public healthcare services by undocumented migrant sex workers (UMSWs) attended in Catalonia between 2013 and 2018. This study utilized a descriptive observational research design. Non-parametric tests were applied to estimate differences in the use of public healthcare services, and incidence and prevalence of selected sexually transmitted diseases (STDs) according to gender and region of origin. Out of 1464 women and 199 men UMSWs, 855 (51.4%) contacted primary care services once or more, and 378 (22.7%) used emergency services. Differences between regions of origin were found in the use of primary care services (Sub-Saharan Africa had 65.9% while Europe and Central Asia 43.0%, *p* < 0.001). Facilitating early diagnosis and improving access to healthcare resources among key populations such as men who have sex with men or transgender women can be achieved through interventions such as community-led point-of-care testing, which increases the frequency of HIV and STD screening and may also prove effective among UMSWs.

## 1. Introduction

In comparison with the general population, female sex workers, transgender sex workers, and male sex workers (SWs) continue to have higher prevalence and incidence rates of sexually transmitted diseases (STDs) and experience limited healthcare access globally, with stark regional disparities, for example, only 39% of female sex workers maintain antiretroviral treatment retention in Cambodia, compared to retention rates of around 70–85% in the general female population; in Uganda, they initiate treatment later than the general population [1]. In Mexico City, 77.7% of male sex workers had never tested for HIV, despite a 38% HIV prevalence. In India, outreach programs have achieved 70% consistent condom use among female sex workers, while in Sub-Saharan Africa, fewer than 50% report regular access to sexual transmitted infection (STI) screening. In Eastern Europe, structural stigma and criminalization result in even lower service use, with coverage of HIV prevention programs falling below 30% in some countries [2,3,4]. According to UNAIDS [5], public health experts [6,7], SW, and human rights organizations [8,9,10], the criminalization of sex work has negative effects on SWs’ access to healthcare.

Ever since the 1948 Declaration of Human Rights, the United Nations has adopted conventions, recommendations, and protocols aiming to eliminate all forms of discrimination. These measures address discrimination based on race, gender, and/or religion, among many others, and include agreements regarding economic, social, cultural, and political rights. The Spanish legislation provides partial regulation of the practice of prostitution by subscribing to human rights treaties relating to freedom, equality, dignity, and safety. In Spain, prostitution is neither legal nor expressly illegal; its practice is not considered a profession, and so it lacks protection in terms of the prevention of occupational risks. For example, the provision of tools for the prevention of STIs or the performance of health check-ups on a regular basis is lacking [11].

Some regions in Spain have approved legislation allowing a type of bar or nightclub where prostitution is practiced, known as “hostess clubs”, as a regulated activity (regulation 217/2002 in Catalonia) [12]. In these clubs, customers pay to spend time with hostesses who engage them in conversation, serve drinks, and entertain them. The Supreme Court of Justice has ruled that there exists an employment relationship in these clubs and thus provides for the protection of hostess club workers; in contrast, this is not the case for those working in brothels (Supreme Court 1084/2016) [13].

Moreover, SWs have to deal with the social stigma that is still linked to sex work. Qualitative studies have reported that sex workers feel ashamed of their occupation or are challenged by family and/or friends [14]. Furthermore, migrants form a heterogeneous group within the community of SWs who may experience particular difficulties in accessing social and health services [15,16], mainly due to linguistic and cultural barriers, or to a lack of familiarity with the healthcare and social system [17]. In addition, SWs are highly vulnerable to HIV and other STDs due to multiple factors, such as having a large number of sexual partners, unsafe working conditions, and impediments to negotiating consistent use of condoms [18,19,20]. In some cases, intravenous drug use may also be an issue [21].

In 2011, a study of 400 SWs carried out in Catalonia, which used structured questionnaires and collected biological samples of saliva and urine, showed that 20.6% of the participants had an STD at some point in their life, that 67% had been in contact with healthcare services at least once in the previous six months, and that 53.4% had at least one voluntary termination of pregnancy [22].

In Catalonia, all residents are granted universal public healthcare coverage by law. Strategies for preventing HIV and STDs in SWs are prioritized, promoting screening for early detection at adapted points-of-care, condom provision, health education, and access to the healthcare system in collaboration with non-governmental organizations (NGOs). In 2005, the Public Health Agency of Catalonia, together with NGOs working to support undocumented migrant sex workers (UMSWs), launched the ‘Functional Plan for access to healthcare in Catalonia for undocumented migrants who practice prostitution’ [23].

To date, no descriptive studies analyzing the health status of UMSWs and their use of public healthcare services have been carried out in Catalonia. Using information from public healthcare administrative registries, the objective of the present study is to examine these issues in the period between 2013 and 2018, and to assess the differences according to region of origin. Among the general population in Catalonia, differences in healthcare use are observed depending on the region of origin [24], and in other studies among SWs in Catalonia (including migrant sex workers) differences in condom use and HIV prevalence were observed depending on region of origin [22].

## 2. Materials and Methods

### 2.1. Study Design and Sampling

A descriptive observational study of UMSWs who applied for health [insurance] cards enabling them to access the public healthcare services (like hospitals and primary care centers) was carried out between 2013 and 2018, during the period of the ‘Functional Plan’.

UMSWs who are in regular contact with NGOs participating in the Functional Plan may apply for a health card even if they are not in possession of a residence or work permit. The health card is associated with a personalized ID, which is simultaneously pseudoanonymized in the healthcare information systems. It is possible to trace the use of public health resources from the data sources described below. It is the responsibility of the Public Health Agency of Catalonia to protect the health of all the population, and especially that of the most vulnerable. Participants in this study were not required to provide informed consent.

The activity within the public health sector of each UMSW was monitored for an entire year since they obtained the health card, as long as their data were not removed from the registry (due to moving to another region or due to death). If an UMSW left Catalonia during the first year (12.3%), the corresponding outcome variables were estimated proportionally for a one-year period.

### 2.2. Data Sources

Data from various administrative registries were used:

− The Working Group on Prostitution and AIDS follow-up registry, which contains the healthcare IDs of all UMSWs in the Functional Plan.
− The Central Registry of Insured People, to obtain the reference population and individual-level information: sex (legal gender as reflected in official document), age, annual income bracket, and benefits received from the Social Security System.
− The Minimum Basic Data Set, an administrative registry containing, at an individual level, all contacts with the public healthcare system: primary care, hospital care, emergency, mental health, and long-term care services. Medical diagnoses are coded using the International Classification of Diseases, 9th and 10th Edition.
− The primary care electronic clinical history program (eCAP) to retrieve visits to specific units: sexual and reproductive healthcare assistance; extractions, tests, and cultures; general practitioners; nursery; medical specialists; emergencies; women’s healthcare assistance; primary care social workers; and other types of visits.

### 2.3. Variables

Dependent variables were:

− The number of people who attended and the number of visits to primary care services, overall and for each unit: sexual and reproductive healthcare assistance; blood tests and microbiological cultures; general practitioners; nursery; medical specialists; emergencies at primary care; women’s healthcare assistance; and other types of visits.
− The number of people who attended and the number of visits to hospital emergency services.
− The prevalence rates of human immunodeficiency viruses (HIV), and human T-lymphotropic virus (HTLV).
− The incidence rates of hepatitis C virus (HCV), hepatitis B virus (HBV), human papillomavirus (HPV), genital herpes, candidiasis, syphilis, molluscum contagiosum, trichomoniasis, chancroid, chlamydia, lymphogranuloma venereum, and gonorrhea (see ICD codes in Table S1).

Independent variables were:

− Sex: male or female.
− Age group: less than 20, 20–24, 25–29, 30–34, and 35 or more.
− Region of origin: Latin America and the Caribbean, Europe and Central Asia, Middle East and North Africa, East Asia, and Pacific and North America.

### 2.4. Statistical Analysis

A descriptive analysis of UMSWs was performed by age group, sex, and SES. Both absolute (N) and relative frequencies (%) were calculated and stratified by region of origin (according to the World Bank classification [24]). Analysis of use of healthcare services (total number N and percentage of attended UMSWs) were calculated. In addition to those, the median and interquartile range, IQR (from weighted number of visits according to the inverse of time in the study in years equivalent) were also carried out for the overall sample. The above-mentioned analysis was followed by a bivariate analysis stratified by region of origin. To determine associations and differences between regions of origin, Chi-square, Fisher’s exact or Kruskal–Wallis tests were performed, according to the type of variable and its distribution, and their *p*-values were included in the results.

Finally, the selected STDs were defined as “chronic” or “non-chronic” depending on the STD being “curable” or not as per WHO definition. Prevalences were calculated for chronic STDs and incidences for non-chronic STDs. HIV and HTLV were considered chronic; while HCV, HBV, HPV, genital herpes, candidiasis, syphilis, molluscum contagiosum, trichomoniasis, chancroid, chlamydia, lymphogranuloma venereum, and gonorrhea were considered non-chronic.

Incidence was calculated as the number of events by 100 person-years. Prevalence was calculated by dividing the number of people with a specific diagnosis by the total number of people at risk and multiplying by 100.

A significance level of 5% was considered in all the tests conducted. All analyses were performed using STATA IC/15.1 software.

## 3. Results

The population studied consisted of 1663 UMSWs, of whom 88.0% were female (1464). The most common region of origin was Latin America and the Caribbean, representing 40.3% (670) of the study population, followed by Europe and Central Asia at 34.0% (565), Sub-Saharan Africa at 23.1% (384), Middle East and North Africa at 1.2% (20), while the least common regions were North America representing 0.1% (1), and East Asia and the Pacific at 1.0% (18). The sex distribution among regions differed; regions where more than 80% of UMSWs were female included Europe and Central Asia, Sub-Saharan Africa, and East Asia and the Pacific, while the rates in the Middle East and North Africa (75.0%), and Latin America and the Caribbean (76.0%) were below 80%. Approximately 55% of the UMSWs were between 20 and 29 years old, while UMSWs from Sub-Saharan Africa were the youngest, given that 66.4% were under 24 years old. Significant overall differences were found between age and region of origin (*p* < 0.001) and between sex and region of origin (*p* < 0.001), but no significant differences between SES and region of origin were found (Table 1 and Table 2).

Nine hundred and fifty-seven (57.3%) women and men contacted one or more primary care or emergency services during their first year since they obtained the health card in Catalonia (51.4% primary care and 22.7% emergency services). The most frequently used primary care services were general practitioner visits (34.5%), followed by blood tests and cultures (32.7%) and sexual and reproductive healthcare assistance (26.5%), while the least used service was meetings with a social worker in a primary care setting (1%). Sexual and reproductive healthcare assistance, general practitioners, and women’s healthcare assistance had a median of two visits per year (IQR: 1–4), while medical specialists and emergency care had a median of one annual visit (IQR: 1–3 and 1–2, respectively) (Table 3). Acute hospitalization, outpatient mental healthcare services, psychiatric services, and long-term care services were not used.

The Sub-Saharan Africa and East Asia and Pacific origin groups of UMSWs had the highest rates of use of primary care (65.9% and 61.1%, respectively), while those with the lowest use were those from Europe and Central Asia (43.0%) and Latin America and the Caribbean (49.6%). Significant differences were found in the performance of blood tests and cultures (ranging from 25.3% in individuals from Europe and Central Asia to 48.4% in those from Sub-Saharan Africa), general practitioner visits (ranging from 26.2% in individuals from Europe and Central Asia to 51.0% in those from Sub-Saharan Africa), nursery care (with attendance rates ranging from 14.2% in individuals from Europe and Central Asia to 42.0% in those from Sub-Saharan Africa), and for women’s healthcare assistance (ranging from 0.89% in those of Latin American and Caribbean origin to 23.4% in those from Sub-Saharan Africa). Only emergency care and primary care assistance from social workers did not present statistically significant differences (Table 4).

Significant differences were observed regarding the median number of visits to the different units according to region of origin: at blood test and culture units, Sub-Saharan Africa, and Latin American and Caribbean UMSWs had a median of two annual visits (IQR: 2–3 and 1–3, respectively), while those of other origins had a median of at most one annual visit. The median number of visits at the general practitioner unit was two per year; UMSWs of Sub-Saharan Africa and East Asia and Pacific origin had a median of three annual visits (IQR: 1–5 and 1–6, respectively) (Table 4).

Regarding emergency services, differences were also found in the percentage of UMSWs who attended, with the highest percentage being observed in individuals from the Middle East and North Africa (45.0%), followed by those from Sub-Saharan Africa and Europe and Central Asia, with values close to 27.0%. Finally, no significant differences were found regarding the number of visits to emergency services by region of origin, whose median ranged from two annual visits (in the cases of Latin America and Caribbean and Middle East and North Africa) to one or zero in all other regions of origin (Table 4).

The STD with the highest prevalence was HIV (prevalence of 3.15%), considered chronic. Other common STDs (all non-chronic) were HCV and HPV, both with an incidence rate of 0.63 per 100 person-years, followed by HBV, candidiasis and syphilis with an incidence rate of 0.42 per 100 person-years, and genital herpes with an incidence rate of 0.21 per 100 person-years. No cases of the following STDs were found: HTLV, molluscum contagiosum, trichomoniasis, chancroid, chlamydia, lymphogranuloma venereum, and gonorrhea (Table 5).

## 4. Discussion

Our findings suggest that just over half of UMSWs attended primary care and around one fifth used emergency services. About a third of UMSWs visited a general practitioner, microbiology and blood testing laboratories, and sexual and reproductive healthcare assistance units (the latter accounting for the largest number of visits). On the other hand, UMSWs did not attend acute care, mental health services, and long-term care services. These results suggest that UMSWs do not use healthcare services as often as legal residents, in whom rates of attendance at primary care and emergency services in adults aged 15 or above were around 80% and 35%, respectively, or as often as other undocumented migrants among whom 61.0% of adults aged 15 or over attended primary care and 27.7% attended emergency services in this period [25]. This suboptimal healthcare use among UMSWs has also been observed in other developed countries, especially among women [26,27].

Significant differences were found in the use of primary care and hospital services according to the region of origin, echoing previous reports in other western countries [14,28]. In our study, Sub-Saharan Africa and East Asia and the Pacific UMSWs made the most frequent use of primary care services, while those from Sub-Saharan Africa made the most use of blood test and culture units, and made the most visits to general practitioners and nursery units. There were also differences in the use of hospital emergency services, attended by almost half of the UMSWs from the Middle East and North Africa.

The most commonly found STD was HIV, with an estimated prevalence of 3.15%, higher than the estimates found in surveillance systems in Catalonia [29]. Incidence of other common STDs such as HCV, HBV, HPV, candidiasis, and syphilis was also non-negligible. The low use of healthcare services and the irregularity of testing for STDs may indicate an underestimation of the prevalence and incidence of these conditions. Early testing for HIV and other STDs is a public health priority among target populations such as SWs [30]. Barriers to access to healthcare services and the lack of prevention efforts in the occupational health setting are issues that must be addressed. For instance, health services, in addition to being available and accessible, must apply the principles of avoidance of stigma and non-discrimination when attending to SWs [31]. Community-led services (interventions designed, delivered, and monitored by, or with, SWs) have demonstrated benefits in terms of HIV outcomes [32], despite the geographical inequality in the access to these services [33]. Point-of-care testing models have demonstrated increased testing frequency and close links to care/initiation of antiretroviral therapy and are likely to contribute to a decrease in HIV incidence rates [34]. In the context of this research, a community-led service for HIV and other STD infections is following the largest European prospective cohort of gay, bisexual, and other men who have sex with men (MSM) and transgender HIV-negative individuals. Its results show that more frequent testing in cases of high exposure (i.e., based on the number of sexual partners with no consistent condom use) can help bring down HIV rates [35].

A high percentage of the UMSWs in our study were under 30 years old, as in previous reports in the literature [36]. There were significant age differences between regions of origin, with Sub-Saharan African UMSWs being the youngest. The largest group were the UMSWs from Latin America and the Caribbean, presumably for linguistic and cultural reasons [37]. The second most common region of origin was Europe and Central Asia, with a notable presence of SWs from Eastern European countries such as Romania. In this case, although linguistic and cultural barriers may be greater than with regard to migrants from Latin America and the Caribbean, the physical proximity between the countries, the lack of geographical barriers, and the possession of a European passport probably account for their strong presence.

One major limitation of the study is the impossibility of ensuring that individuals who appear in the registries with an assigned birth sex (male or female) do not in fact identify themselves as cisgender, transgender, or non-binary. This lack of information about individuals’ identities limits the capacity of this study to properly analyze this issue.

The decision to use a one-year period of study was taken due to the high mobility among UMSWs, who are likely to leave and/or return to Catalonia quite frequently over a longer period. Studying a five-year period, for example, would have increased the chance of analyzing misleading data recorded after they have ceased sex work or stopped contact with the NGO involved in the Functional Plan. Studying the first year since they obtained the health card in Catalonia was agreed to be the best solution to this problem in order to make data periods as homogeneous as possible for all UMSWs.

Further, the utilization of health services as well as the prevalence and incidence rates of STDs may present an estimation bias, since not all UMSWs contacted the public healthcare system. Possibly, then, the data may not be generalizable to the entire population of UMSWs.

In addition, the lack of morbidity data (i.e., general health status) as well as cultural patterns (knowledge regarding healthcare systems and their cultural appropriateness) limit the understanding of why hospital utilization varies among UMSWs from different regions. Finally, it would have been interesting to link the data from the registry of voluntary termination of pregnancy in order to calculate its absolute and relative rates. However, the individual identifier in this registry is the medical history code, which cannot be directly linked to other healthcare ID codes.

## 5. Conclusions

The use of the administrative healthcare records provides new insights into the situation of the UMSW community. In fact, in all likelihood this is the first time some information, such as the use of healthcare services by UMSWs, has been reported in Catalonia. Studies with data from population-based administrative registries are extremely valuable since they allow the recording of information for large samples, obtaining results in a relatively short period of time, and requiring fewer resources than surveys. This approach also overcomes certain survey-based study limitations, as the migrant SWs surveyed may not provide accurate answers when asked about the number of visits or their health outcomes over the past twelve months, due to potential recall bias.

The results regarding UMSWs healthcare use, and in relation to the different regions of origin in particular, will allow policymakers to focus on ways of reducing inequalities in the use of healthcare services for primary and secondary prevention and health promotion. Nonetheless, it would be desirable to increase the statistical power in order to ascertain whether the observable differences among the various origins are truly meaningful rather than attributable to chance, ideally through the promotion of multicenter studies conducted in socio-economic, healthcare, and political contexts comparable to that of Spain.

The lack of regulation in relation to the workplace and, consequently, the lack of preventive measures in the field of occupational health represents structural barriers for those needing to access healthcare services. Without explicit regulations, it is difficult to meet the health needs of SWs. Community-led services that ensure regular testing, promote health, and prevent new infections in UMSWs could be part of the solution, as observed in studies of other risk groups for STDs.

## Figures and Tables

**Table 1:** Population characteristics.

Variables	Total	
	N	%
Total	1663	100.0
Age group ^a^		
Less than 20 years	170	10.2
20–24	512	30.8
25–29	403	24.2
30–34	259	15.6
35 or more years	319	19.2
Sex ^a^		
Female	1464	88.0
Male	199	12.0

^a^ Fisher’s Exact Test.

**Table 2:** Population characteristics by region of origin.

Variables	Latin America and Caribbean		Europe and Central Asia		Middle East and North Africa		Sub-Saharan Africa		Asia and the Pacific		North America		*p*-Value
	N	%	N	%	N	%	N	%	N	%	N	%	
Total	675	40.6	565	34.0	20	1.2	384	23.1	18	1.0	1	0.1	
Age group ^a^													
Less than 20 years	21	3.1	73	12.9	1	5.0	73	19.0	2	11.1	0	0.0	<0.001
20–24	149	22.1	177	31.3	3	15.0	182	47.4	1	5.6	0	0.0	
25–29	196	29.0	134	23.7	2	10.0	67	17.4	3	16.7	1	100.0	
30–34	130	19.3	84	14.9	6	30.0	39	10.2	0	0.0	0	0.0	
35 or more years	179	26.5	97	17.2	8	40.0	23	6.0	12	66.7	0	0.0	
Sex ^a^													
Female	514	76.15	541	95.75	15	75.00	379	98.70	15	83.33	0	0.00	<0.001
Male	161	23.85	24	4.25	5	25.00	5	1.30	3	16.67	1	100	

^a^ Fisher’s Exact Test.

**Table 3:** Use of healthcare services.

	Total	
	N	%
Individuals attended at primary care, specialist units, or hospital emergency services (%)	953	57.3
Individuals attended at primary care or specialist units (%)	N	%
Overall	855	51.4
Sexual and reproductive healthcare assistance	441	26.5
Extractions, tests, and cultures	544	32.7
General practitioner	573	34.5
Nursery	364	21.9
Medical specialist	128	7.7
Emergency	106	6.4
Women’s healthcare assistance	128	7.7
Social worker assistance	26	1.6
Other types of visits	51	3.07
Mean primary care or specialist unit visits (Median (IQR))	N	Median (IQR)
Sexual and reproductive healthcare assistance	1266	2 (1–4)
Extractions, tests, and cultures	1105	2 (1–3)
General practitioner	1856	2 (1–4)
Nursery	807	1.71 (1–3)
Medical specialist	305	1 (1–3)
Emergency	177	1 (1–2)
Women’s healthcare assistance	387	2 (1–4)
Social worker assistance	48	1.5 (1–2)
Other types of visits	122	1 (1–2)
Individuals attending hospital emergency services (%)	378	22.73%
Mean hospital emergency visits (mean (sd))	808	1 (1–2)

**Table 4:** Use of healthcare services by region of origin.

	Latin America and Caribbean		Europe and Central Asia		Sub-Saharan Africa		Middle East and North Africa		East Asia and the Pacific		North America		*p*-Value
	N	%	N	%	N	%	N	%	N	%	N	%	
Attended at primary care, specialist units or hospital emergency services	363	53.78	295	52.21	268	69.79	14	70.00	12	66.67	1	100.00	<0.001
Individuals attending primary care or specialist units													
Overall	335	49.63	243	43.01	253	65.89	11	55.00	11	61.11	1	100.00	<0.001
sexual and reproductive healthcare ^a^	199	29.48	118	20.88	115	29.95	4	20.00	5	27.78	0	0	0.004
Extractions, tests, and cultures ^a^	204	30.22	143	25.31	186	48.44	4	20.00	7	38.89	0	0	<0.001
General practitioner ^a^	210	31.11	148	26.19	196	51.04	10	50.00	8	44.44	1	100.00	<0.001
Nursery ^a^	109	16.15	80	14.16	161	41.93	7	35.00	7	38.89	0	0	<0.001
Medical specialist ^a^	42	6.22	45	7.96	32	8.33	5	25.00	4	22.22	0	0	0.011
Emergency ^a^	43	6.37	27	4.78	31	8.07	2	10.00	3	16.67	0	0	0.091
Women’s healthcare assistance ^a^	6	0.89	32	5.66	90	23.44	0	0.00	0	0.00	0	0	<0.001
Social worker assistance ^a^	9	1.33	11	1.95	6	1.56	0	0.00	0	0.00	0	0	0.850
Other types of visits ^a^	19	2.81	10	1.77	20	5.21	2	10.00	0	0.00	0	0	0.021
Visits at primary care or specialist units	N	Median (IQR)	N	Median (IQR)	N	Median (IQR)	N	Median (IQR)	N	Median (IQR)	N	Median (IQR)	
Sexual and reproductive healthcare ^b^	512	2 (1–3)	364	2 (1–4)	371	2 (1–4)	7	1.5 (1–2.5)	11	2 (1–3)	0	**	0.385
Extractions, tests, and cultures ^b^	386	2 (1–2)	281	1 (1–2)	419	2 (1–3)	8	1.5 (1–3)	11	1 (1–2)	0	**	0.049
General practitioner ^b^	559	2 (1–4)	509	2 (1–4)	702	3 (1–5)	47	2 (1–6)	38	3 (2–5)	1	1 (1–1)	0.003
Nursery ^b^	217	1 (1–2)	190	1.2 (1–3)	372	2 (1–3)	15	1 (1–2)	13	2 (1–3)	0	**	0.664
Medical specialist ^b^	90	1 (1–2)	113	2 (1–3)	77	1 (1–2)	14	3 (1–3)	11	1.5 (1–4.5)	0	**	0.642
Emergency ^b^	71	1 (1–2)	53	1 (1–3)	43	1 (1–1)	7	3.5 (2–5)	3	1 (1–1)	0	**	0.053
Women’s healthcare assistance ^b^	10	1.5 (1–2)	89	2 (1–3)	288	2 (1–4)	0	**	0	**	0	**	0
Social worker assistance ^b^	14	1 (1–2)	16	1 (1–2)	18	2.5 (2–5)	0	**	0	**	0	**	0.056
Other types of visits ^b^	54	1 (1–3)	23	2 (1–3)	42	1.5 (1–2)	3	1.5 (1–2)	0	**	0	**	0.895
Individuals attending hospital emergency services (N,%) ^a^	107	15.85	151	26.73	105	27.34	9	45.00	4	22.22	0	0.00%	<0.001
Hospital emergency visits (N, Median (IQR)) ^b^	217	2 (1–2)	323	1 (1–3)	229	1 (1–2)	34	2 (1–4)	5	1 (1–1.5)	0	**	0.615

^a^: Kruskal–Wallis; ^b^: Fisher’s Exact Test; ** This value cannot be computed.

**Table 5:** Prevalence and incidence density of selected sexually transmitted diseases.

	Total	
	N	%
Prevalence		
Human Immunodeficiency Virus	30	3.15%
Human T-lymphotropic virus	0	0.00%
Incidence density		py *
Hepatitis C virus	6	0.63
Hepatitis B virus	4	0.42
Human papilloma virus	6	0.63
Genital herpes	2	0.21
Candidiasis	4	0.42
Syphilis	4	0.42
Molluscum contagiosum	0	0.00
Trichomoniasis	0	0.00
Chancroid	0	0.00
Chlamydia	0	0.00
Lymphogranuloma venereum	0	0.00
Gonorrhea	0	0.00

* py: By 100 person-years.

## Data Availability

The treatment of clinical and health data by AQuAS is regulated by the Organic Law 3/2018, of 5 December, on the protection of personal data and guarantee of digital rights (LOPDGDD); the General Data Protection Regulation (RGPD) (EU) 2016/679, of 27 April; as well as the considerations issued by the Catalan Data Protection Authority (APDCAT). In all cases, the reuse of clinical and health data of natural persons is allowed on epidemiological and clinical grounds and for the purposes of health research purposes. In this study, a pseudo-anonymization process of the personal identification code called NIA was used.

## Supplementary Materials

The following supporting information can be downloaded at: https://www.mdpi.com/article/10.3390/ijerph22111696/s1, Table S1: Selected sexually transmitted diseases.

## Abbreviations

The following abbreviations are used in this manuscript:

AIDS	Acquired Immune Deficiency Syndrome
POC	Point-of-Care
HIV	Human Immunodeficiency Virus
HTLV	Human T-lymphotropic Virus
HPV	Human Papillomavirus
HBV	Hepatitis B Virus
HCV	Hepatitis C Virus
STD	Sexually Transmitted Diseases
UMSW	Undocumented Migrant Sex Worker
NGOs	Non-Governmental Organizations
ICD	International Classification of Diseases
IQR	Interquartile Range
UNAIDS	The Joint United Nations Programme on HIV/AIDS
NIA	Anonimised ID Number
AQuAS	Catalan Agency for Health Quality and Assessment of Catalonia
CIBERESP	CIBER de Epidemiología y Salud Pública
PCAVIHV	Programa de Prevenció, Control i Atenció al VIH, les ITS i les Hepatitis Víriques
IIB Sant Pau	Institut d’Investigació Biomèdica
eCAP	primary care electronic clinical history program
CEIM	Comitè d’Ètica de la Investigació amb medicaments (Ethics Committee for Research with Medicines)
IDAP	Institut Universitari per a la recerca a l’Atenció Primària de Salut Jordi Gol i Gurina
