# Supplementary material for: Health Status and Use of Healthcare Services of Undocumented Migrant Sex Workers in Catalonia: A Descriptive Study Using Administrative Registries
Source: Int J Environ Res Public Health. 2025 Nov 10;22(11):1696. doi: 10.3390/ijerph22111696 (PMC12652567; doi:10.3390/ijerph22111696)
Supplement: Supplementary file 1 [file ijerph-22-01696-s001.zip › ijerph-3827837-supplementary.pdf]

Table S1. Selected sexually transmitted diseases.

STD[a] tag name	Prevalence / Incidence	ICD-9 code	ICD-10 code
HIV[b]	Prevalence of Human Immunodeficiency Virus (HIV) infection	042, 043, 044 and 079.53	B20, B21, B22, B23, B24, Z21 and O98.7
HCV	Incidence of Hepatitis C Virus (HCV) infection	070.7	B17.1 and B18.2
HBV	Incidence of Hepatitis B Virus (HBV) infection	070.2 and 070.3	B16, B17.0, B18.0 and B18.1
HPV	Incidence of Human Papilloma Virus (HPV) infection	078.10, 078.11, 078.19 and 079.4	B97.7, R85.81, R87.81, R85.82 and R87.82
HTLV[b]	Prevalence of Lymphotropic Virus Infection of Human T Cells (1 and 2)	079.51 and 079.52	B97.33, Z22.6 and B97.34
Genital herpes	Incidence of *Herpesvirusc sp*. to anogenital areas	054.10, 054.11, 054.12 and 054.19	A60
Molluscum contagiosum	Incidence of *Molluscipoxvirus* infection	078.0	B08.1
Candidiasis	Incidence of *Candida sp*. to anogenital areas	112.1 and 112.2	B37.3 and B37.4
Chlamydia	Incidence of *Chlamydia trachomatis* infection in anogenital areas	099.41, 099.52, 099.53 and 099.56	A56
LGV	Incidence of *Lymphogranuloma venereum* (LGV) infection	099.1	A55
Gonorrhea	Incidence of *Neisseria gonorrhoeae* infection in anogenital areas	098.0, 098.1, 098.2, 098.3 and 098.7	A54.0, A54.1 and A54.2
Syphilis	Incidence of *Treponema pallidum subsp. pallidum* in anogenital areas	091.0, 091.1, 091.2 and 091.3	A50, A51, A52 and A53
Trichomoniasis	Incidence of *Trichomonas vaginalis* infection	131.0 and 007.3	A59
Chancroid	Incidence of *Haemophilus ducreyi* infection in anogenital areas	099.0	A57
